# Importance of the Selected Cut-Offs for Serum Uric Acid and Lipids Levels

**DOI:** 10.1155/2014/746561

**Published:** 2014-05-14

**Authors:** Huseyin Kayadibi, Erdim Sertoglu, Metin Uyanik

**Affiliations:** ^1^Biochemistry Laboratory, Department of Medical Biochemistry, Adana Military Hospital, 01150 Adana, Turkey; ^2^Biochemistry Laboratory, Ankara Mevki Military Hospital, Anittepe Dispensary, Ankara, Turkey; ^3^Department of Medical Biochemistry, Gulhane School of Medicine, Ankara, Turkey

We read with great interest the recently published article “*The prevalence of nonalcoholic fatty liver disease and relationship with serum uric acid level in Uyghur population*” by Cai et al. [1]. Authors have investigated the prevalence of nonalcoholic fatty liver disease (NAFLD) and the association of serum uric acid (SUA) level with NAFLD in Uyghur people. They concluded that SUA level was significantly associated with NAFLD, and the prevalence rate of NAFLD increased with progressively higher SUA levels. However, we think that there are some points that should be emphasized about this study.

Firstly, the cut-off value for fasting plasma glucose (FPG) was taken as 6.10 mmol/L in Table 5, while it was determined as 5.6 mmol/L in the section Diagnostic Criteria. Due to this difference, *P* value, determined by multivariate logistic regression analysis, may vary and even become meaningless while it was statistically significant.

Secondly, since total cholesterol (TC) is defined as the sum of HDL-C, LDL-C, and VLDL-C, it has to be technically higher than LDL-C. However, in Table 5 the same regression cut-off values were used for TC and LDL-C. This improper preference may lead to incorrect results in regression analysis. In addition, as can be seen in Table 6, authors included three lipid parameters (TC, TG, and HDL-C) into the multivariate logistic regression analysis. However, since these parameters are tightly correlated with each other, results, especially odds ratio and Wald value, can easily be affected. Adding or removing only a single parameter to multivariate logistic regression analysis will change all of the results, due to the relative effect of each parameter.

Thirdly, in many studies, different diagnostic criteria for hyperuricemia were used for males (>7 mg/dL or >420 *μ*mol/L) and females (>6 mg/dL or >360 *μ*mol/L), as indicated in the original study [2–4]. However, this distinction was not taken into consideration when comparing the NAFLD group with non-NAFLD group in Table 2. 42.8% of non-NAFLD and 72.4% of NAFLD patients were male. This big difference of 29.6% may be the reason for the statistical significance. When patients were divided according to the gender, this significance may not be seen.

In conclusion, in multivariate logistic regression analysis included and removed parameters are highly important since the relative effect of each parameter is calculated in this analysis. It is also important to use the gender specific reference ranges for SUA.
